# Influence of Tea Tree Essential Oil and Poly(ethylene glycol) on Antibacterial and Physicochemical Properties of Polylactide-Based Films

**DOI:** 10.3390/ma13214953

**Published:** 2020-11-04

**Authors:** Iwona Tarach, Ewa Olewnik-Kruszkowska, Agnieszka Richert, Magdalena Gierszewska, Anna Rudawska

**Affiliations:** 1 Chair of Physical Chemistry and Physicochemistry of Polymers, Faculty of Chemistry, Nicolaus Copernicus University in Toruń, Gagarina 7 Street, 87-100 Toruń, Poland; tarach@doktorant.umk.pl (I.T.); mgd@umk.pl (M.G.); 2 Chair of Genetics, Faculty of Biological and Veterinary Sciences, Nicolaus Copernicus University in Toruń, Lwowska 1 Street, 87-100 Toruń, Poland; a.richert@umk.pl; 3 Department of Production Engineering, Faculty of Mechanical Engineering, Lublin University of Technology, 20-618 Lublin, Poland; a.rudawska@pollub.pl

**Keywords:** polylactide, tea tree essential oil, poly(ethylene glycol), packaging material, antibacterial films

## Abstract

The aim of the study was to establish the influence of poly(ethylene glycol) (PEG) on the properties of potential biodegradable packaging materials with antibacterial properties, based on polylactide (PLA) and tea tree essential oil (TTO). The obtained polymeric films consisted of PLA, a natural biocide, and tea tree essential oil (5–20 wt. %) was prepared with or without an addition of 5 wt. % PEG. The PLA-based materials have been tested, taking into account their morphology, and their thermal, mechanical and antibacterial properties against Staphylococcus aureus and Escherichia coli. It was established that the introduction of a plasticizer into the PLA–TTO systems leads to an increase in tensile strength, resistance to deformation, as well an increased thermal stability, in comparison to films modified using only TTO. The incorporation of 5 wt. % PEG in the PLA solution containing 5 wt. % TTO allowed us to obtain a material exhibiting a satisfactory antibacterial effect on both groups of representative bacteria. The presented results indicated a beneficial effect of PEG on the antibacterial and functional properties of materials with the addition of TTO.

## 1. Introduction

The proliferation of bacteria contributes to the spread of diseases, as well as food spoilage [1,2]. This group of microorganisms includes mainly strains such as *Bacillus cereus*, *Listeria monocytogenes*, *Salmonella typhimurium* and *Staphylococcus aureus* [3,4]. Due to the possibility of the undesirable contact of microorganisms with food, effective and safe methods of combating pathogenic bacteria are being sought [5]. For this reason, the food industry is still looking for new, innovative solutions to combat microorganisms’ proliferation in packaged products, and to extend their shelf-life [6].

The manufacturing of packaging with antibacterial properties relies on the direct incorporation of biologically active substances into the polymer matrix [7]. In particular, the application of natural antibacterial agents for this purpose is noteworthy [8]. Essential oils (EO) are natural, microbiologically active substances with an extensive spectrum of activity [9]. Due to the acceptance of PLA and EO by the American Food and Drug Administration as “Generally Recognized As Safe GRAS”, a significant development of studies of materials based on the above-mentioned compounds has taken place [10]. Ahmed et al. in their works described the results of detailed research devoted to films based on polylactide (PLA) with the addition of cinnamon oil [11,12]. The application of other essential oils, such as clove [4,13], garlic [9,12], as well as lemongrass, rosemary and bergamot essential oil [14], allowed the obtaining of PLA-based materials displaying antibacterial properties.

Tea tree oil (TTO) belongs to the group of essential oils exhibiting bactericidal properties. It is obtained during the distillation of *Melaleuca alternifolia* leaves [15]. TTO contains a mixture of terpenes (monoterpenes and sesquiterpenes) and their associated tertiary alcohols [16]. This essential oil is characterized by the capacity to inhibit the development of various microorganisms. As a result, it can be applied to effectively combat different types of respiratory and oral cavity infections, as well as skin diseases, and supports wound healing [5]. It should be stressed that TTO also exhibits anti-inflammatory, antiviral, antioxidant, antiseptic and antifungal properties [10]. The antibacterial potential of tea tree essential oil is mainly related to the presence of terpinen-4-ol and 1,8-cineole [8]. The other components of TTO synergistically intensify the antibacterial effect of individual compounds in the mixture, and ensure the low probability of bacterial resistance to the biocide in question [4]. The mechanism of combating microorganisms with tea tree essential oil is not yet fully understood. It is, however, known that TTO strongly interacts with the bacterial cell membrane and disrupts its vital functions [5]. The effectiveness of TTO has contributed to the extensive application of this substance in the cosmetics and pharmaceutical industries, as well as in medicine [16]. Moreover, publications devoted to the incorporation of TTO into the polymer matrix indicate the possibility of obtaining materials with satisfactory antibacterial properties [8,17,18]. It is noteworthy that PLA-based films with the addition of TTO have not been described in the literature so far.

Plasticizers are often used in order to increase the elasticity of polymer films [12]. Poly(ethylene glycol), poly(propylene glycol), lactic acid, and other polymers miscible with PLA count among the most common substances used to plasticize polylactide [9]. PEG is widely used, in particular due to its biodegradability. There are several works describing the characteristics of PLA-based materials with the addition of various essential oils [14], or PLA/PEG blends modified with these biocides [12,19]. Nevertheless, the influence of the plasticizer on the properties of a neat PLA film, as well as on PLA materials modified with tea tree essential oil, has not been studied yet.

The conducted research includes the development of new potential packaging films based on PLA and TTO, both with and without the addition of PEG as a plasticizer. The obtained films were characterized in terms of structure, as well as their antibacterial, mechanical and thermal properties. The novelty of the study was in determining the effect of the plasticizer on the functional properties, and the antibacterial effects against the *Staphylococcus aureus* and *Escherichia coli* strains, of PLA-based films modified with tea tree essential oil.

## 2. Materials and Methods

### 2.1. Materials

Polylactide (PLA) 2002D type (NatureWorks^®^, Minnetonka, MN, USA) with a melt flow rate of 5–7 g 10 min^−1^ (2.16 kg; 190 °C) and a density of 1.24 g cm^−3^ in the form of pellets was used to prepare polymer solutions. Tea tree essential oil (TTO) obtained from the leaves of Melaleuca alternifolia was purchased from Sigma-Aldrich Ltd., Poland. Poly(ethylene glycol) (PEG) with M_w_ = 1 500 g mol^−1^ (Sigma-Aldrich Ltd., Poznan, Poland) was used as a plasticizer. Chloroform (manufactured by Chempur, Piekary Śląskie, Poland) was used as a solvent.

### 2.2. Preparation of Films

The examined films were prepared using the solvent-casting method. For this purpose, polylactide pellets were dissolved in chloroform in an attempt to obtain a 3% (w/v) polymer solution. Subsequently 5 wt. %, 10 wt. % or 20 wt. % tea tree essential oil was added to the PLA solution (relative to the weight of polylactide used). In total, 5 wt. % PEG was introduced into the solutions to prepare plasticized PLA-based films. In order to obtain PLA-based materials, 50 mL of the prepared mixture was poured onto glass Petri dishes (14.5 cm in diameter) and left for 3 days to form a polymer film.

The film marked as L consists of neat polylactide. The PLA-based material containing 5 wt. % PEG was named as LP. The LTx symbol indicates samples modified only with tea tree essential oil (where x denotes the content of the biocide in relation to the mass of polylactide used to form a particular sample). Films with the addition of both PEG and 5 wt. %, 10 wt. % or 20 wt. % TTO were designated as LPT05, LPT10 and LPT20, respectively.

### 2.3. Methods of Analysis

#### 2.3.1. Scanning Electron Microscopy

The morphology of the PLA-based films was studied by means of the Quanta 3D FEG scanning electron microscope (SEM, FEI Company, Hillsboro, OR, USA). Photographs of the topography of the samples were taken using the SE detector in 10,000-fold magnification. Before each of the analyses, the surfaces of the studied materials were sprayed with a layer of gold.

#### 2.3.2. Atomic Force Microscopy

The roughness analyses of the obtained PLA materials, modified with TTO or TTO with a PEG addition, employed an atomic force microscope (AFM, NanoScope MultiMode, Veeco Metrology, Inc., Santa Barbara, CA, USA). Surface images of the studied films were obtained using an AFM microscope with an SPM scanning probe of the NanoScope MultiMode type (Veeco Metrology, Inc. Santa Barbara, CA, USA) in tapping mode. All analyses were conducted in air, at room temperature. Roughness parameters were calculated by applying the Nanoscope software for sample areas measuring 5 μm × 5 μm.

#### 2.3.3. Thermogravimetry

Analyses of thermal stability of the PLA-based films were carried out by means of a Simultaneous TGA-DTA thermal analyzer of the SDT 2960 type (TA Instruments, London, UK) in the range of room temperature to 600 °C. All measurements were performed at a heating rate of 10 °C/min in an air stream.

#### 2.3.4. Differential Scanning Calorimetry

The thermal properties (temperatures and enthalpy changes of individual phase transitions) of the studied materials were determined using a differential scanning calorimeter (Polymer Laboratories Ltd., Epsom, UK). Measurements were taken in the range of 30 to 180 °C, with a heating rate of 10 °C/min under nitrogen. On the basis of the obtained data, the degree of crystallinity ($X_{c}$) of the PLA-based materials was also calculated according to the following Formula (1):

(1) $$X_{c}=\frac{∆H_{m}^{PLA}-∆H_{c}^{PLA}}{∆H_{}^{o}\cdot X_{PLA}}\cdot 100\%$$

where $∆H_{m}^{PLA}$ is a change of melting enthalpy, $∆H_{c}^{PLA}$ is a change of enthalpy of cold crystallization, and $X_{PLA}$ was marked as the mass fraction of polylactide in the tested sample. $∆H_{}^{o}$ indicates the change of melting enthalpy of 100% crystalline PLA, the value of which, according to Sosnowski’s work [20], was predetermined as 109 J/g.

#### 2.3.5. Uniaxial Tensile Test

The examination of the mechanical properties of the obtained PLA-based films was performed using the Intron 1193 machine in accordance with the ISO 527-1:2020 [21] and ISO 527-3:2019 [22] standards. Paddle-shaped samples were stretched at a speed of 0.50 cm/min by means of the crosshead with an applied 50 N force. At least five measurements were made in the case of each of the studied films. Based on obtained data, the value percentage of the elongation at break and tensile strength were determined. The values of the Young’s modulus were calculated from the rectilinear region of the registered curve during the stretching of samples.

#### 2.3.6. Thickness and Transparency of Studied Materials

The thickness of the obtained materials was established as the average of five measurements performed using an Absolute Digimatic Indicator, Sylvac S229 Swiss (Yverdon, Switzerland), with a precision of 0.001 mm. In order to determine the transparency of the PLA-based films, transmittance at 600 nm (*T*_600_) was measured by means of a UV spectrophotometer (Ruili Analytical Instrument Company, Beijing, China). The samples were cut into rectangles and placed in a spectrophotometer test cell. An empty cuvette was used as the blank. The transparency value (*TV*) of the PLA-based materials was calculated according to Equation (2) presented in the work of Arfat [19]:

(2) $$TV=\frac{-logT_{600}^{}}{x}$$

where *x* is the film thickness (mm). Moreover, it should be stressed that a higher transparency value (*TV*) indicates the lower transparency of the studied materials.

#### 2.3.7. Evaluation of Antibacterial Activity

In order to evaluate the antibacterial properties of the PLA-based films with an addition of TTO or TTO and a plasticizer, a disk-diffusion method was applied in accordance with the ISO 20645:2006 standard [23]. Agar plates were inoculated with representative strains of *Staphylococcus aureus* (ATCC 6538P) or *Escherichia coli* (ATCC 8739), and a bacteria concentration of 1.5 × 10^8^ CFU/mL. Samples with a diameter of 25 ± 5 mm were placed onto an inoculated agar and incubated for 20 h at 37 ± 1 °C.

The size of the inhibition zones of bacterial growth, as well as the degree of microorganism proliferation in the direct contact between the studied films and agar, were examined using an Olympus SZX 12 stereoscopic microscope. Photographs of the examined materials were taken using an ARTRAY camera (ARTCAM 300MI model) in a 60-fold magnification mode. The appearances of the samples and agar plates after the investigated films had been removed was recorded by means of a SCAN^®^ 1200 colony counter (Interscience, Saint-Nom-la-Bretèche, France).

## 3. Results and Discussion

### 3.1. Assessment of Film Morphology

**Figure 1:**
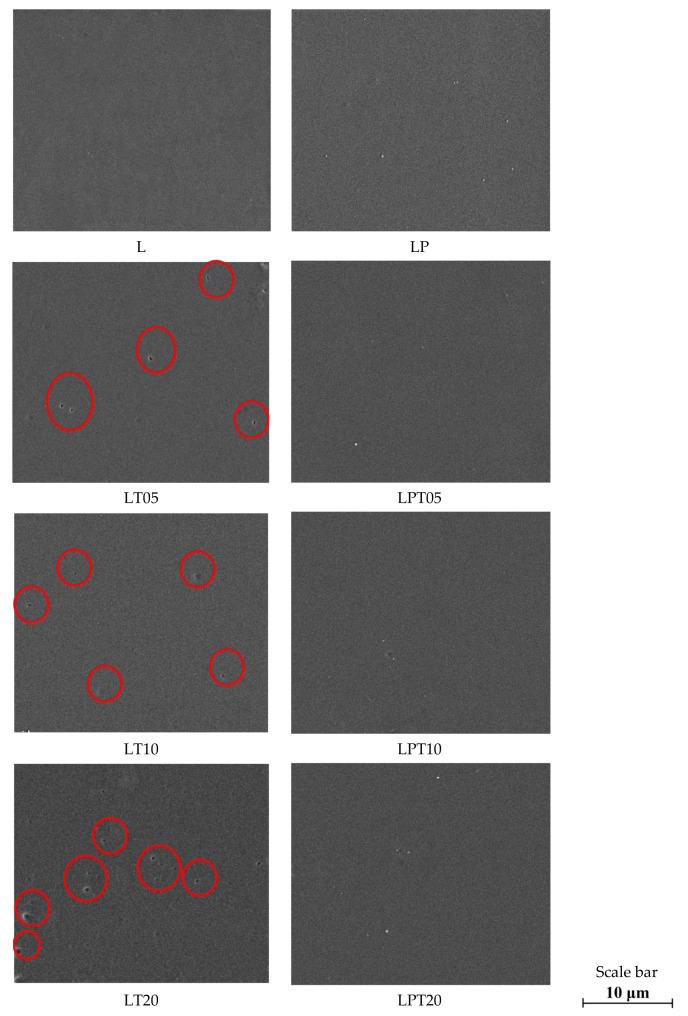
Structure of plasticized or unplasticized films based on polylactide (PLA) with the addition of different concentrations of TTO (magnification 10,000×).

The morphology of the PLA-based films was studied by means of scanning electron microscopy (Figure 1). The material consisting entirely of polylactide was characterized by a homogeneous and smooth surface. The PLA-based films with an addition of tea tree essential oil had cavities and holes which formed an irregular surface, similar to materials with an addition of oregano essential oil, as described in Javidi’s work [24]. The materials described by Javidi et al., however, exhibited a less uniform structure than the PLA-TTO films. This can be attributed to the difference between the content and the extent of volatility displayed by TTO and the essential oil used in the mentioned work [24]. Moreover, PLA-based materials modified with tea tree essential oil had significantly fewer pores in their surface compared to the films based on gelatin with an addition of clove essential oil, as characterized by Ejaz [13]. The size as well as the number of pores on the surface of the obtained materials increased with the rise in the amount of TTO introduced into the PLA matrix (Figure 1). The factor which significantly affected the morphology of the films consisting of polylactide and tea tree essential oil was the partial volatilization of the biocide during the formation of the studied materials. Moreover, changes in the external structures of the PLA films modified only with TTO also resulted from the presence of the biocide droplets embedded between polymer chains. This mentioned effect was described in Qin’s work [14] devoted to the formation of materials based on PLA and bergamot, lemongrass, rosemary and clove essential oils.

**Figure 2:**
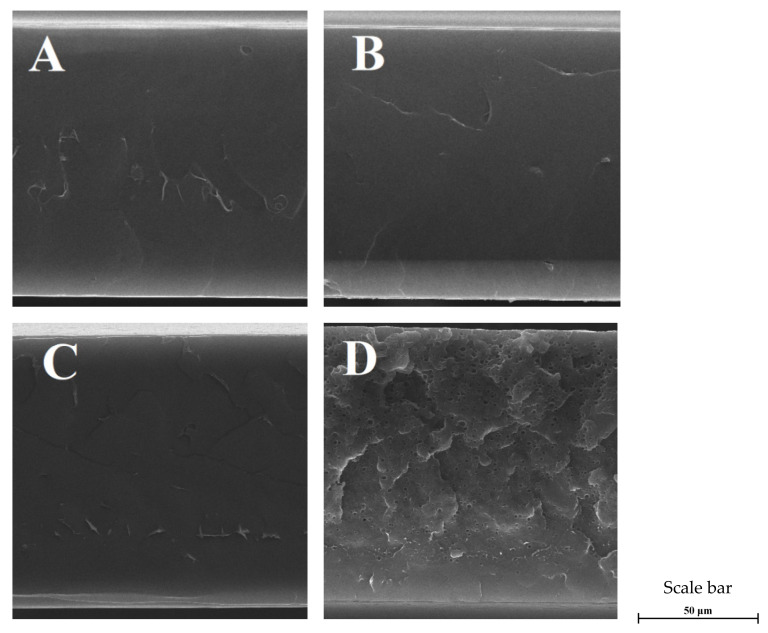
Cross-sections of films PLA modified with (**A**) 5%, (**B**) 10%, (**C**) 20% and (**D**) 50% TTO.

On the other hand, the incorporation of the plasticizer into a neat PLA results in only a slight irregularity of the film surface (LP sample Figure 1). This is related to the phase separation of the PLA–PEG mixture during chloroform evaporation and the formation of small aggregates of plasticizer inside the polylactide matrix [25]. SEM photographs of the plasticized materials, based on polylactide with the addition of TTO, reveal convexities associated with the presence of PEG, which does not uniformly blend with PLA [26]. Moreover, in the cases of samples consisting of PLA, PEG and TTO pores were not formed while the materials were being dried. Based on the obtained results, it can be assumed that a reduction in the interactions between the PLA chains and PEG caused by the introduced molecules of TTO allows for preventing the flocculation and coalescence of essential oil during the formation of films. The magnitude of interactions between TTO and PLA plays a crucial role during the evaporation of the organic solvent [24]. For this reason, an insufficiently strong interaction between molecules of the essential oil and the polymer leads to the phase separation of this mixture. Therefore, it is justified to assume that the PLA and TTO mixtures without the addition of PEG during evaporation of chloroform may undergo the processes characteristic of two-phase dispersion systems, despite the solubility of the polymer and the essential oil in the mentioned solvent [8]. In order to confirm the occurrence of the flocculation, coalescence and creaming phenomena during the drying of the PLA-based films modified solely with TTO, SEM analyses of the cross-sections of all the tested materials were carried out (Figure 2).

In the cases of films without the addition of PEG containing from 5% to 20% TTO, a compact structure of films was observed without the presence of pores in the materials. This leads to the conclusion that due to the high volatility of the incorporated biocidal substance, the migration of droplets of essential oil onto the surfaces of the materials did not result in the formation of a discontinuous structure. In order to verify that the creaming of the PLA–TTO mixtures containing higher amounts of biocidal substance during their drying had taken place, a PLA-based film with an addition of 50 wt. % tea tree essential oil was also imaged. Studies of that sample showed both the coalescence and the creaming of biocide droplets inside the PLA matrix during the evaporation of the solvent (Figure 2) [18,27]. These observations indicate the necessity of using essential oils in an amount less than 50%, in order to avoid the premature release of some volatile biocides from the polylactide film.

### 3.2. Examination of Surface Topography of PLA-Based Films

**Figure 3:**
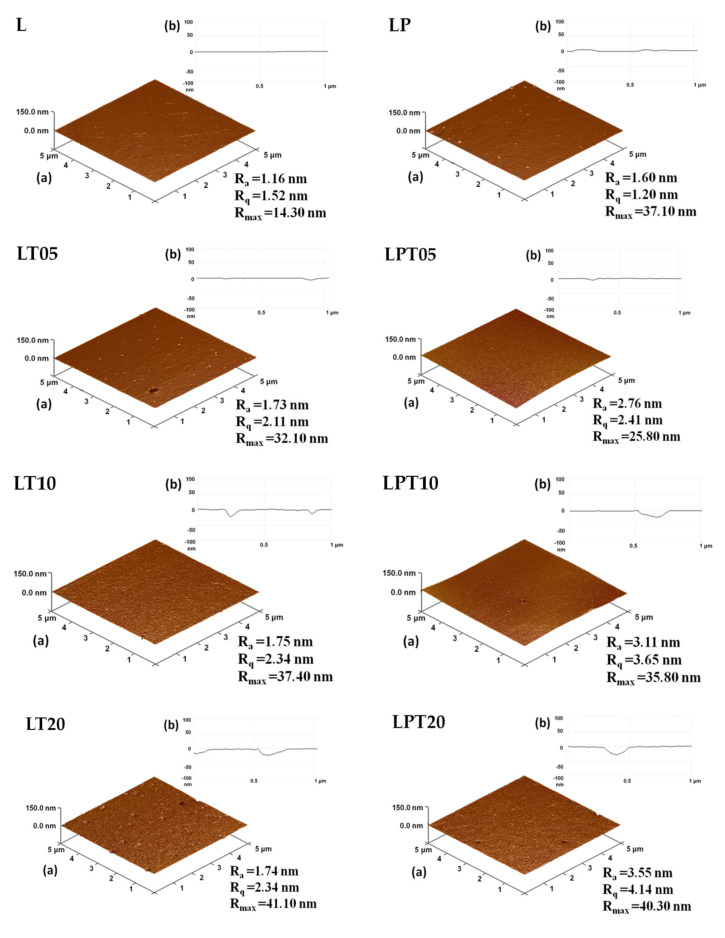
Roughness of PLA-based films ((**a**) 3D imaging and (**b**) cross-sections).

The capacity of materials to combat the proliferation of bacteria on their surface results from the presence of biologically active substances [28]. Moreover, in the case of packaging films, the effectiveness of the biocides introduced into the polymer matrix is affected by the structure of these materials—mainly by the actual surface of direct contact with the bacteria cells. In order to assess the roughness of the polymer, the film analyses carried out using AFM microscopy are extremely valuable [29]. Figure 3 depicts the values of the R_a_ (mean arithmetic deviation of the profile from the mean line), R_q_ (mean square deviation of surface roughness) and R_max_ (maximum distance between the highest and lowest points of the recorded image) parameters for the studied samples. The recorded AFM images indicate that due to the incorporation of tea tree essential oil into the PLA matrix, the surfaces of the films became more porous in comparison to the smooth surface of the control sample (pure PLA (Figure 3)). Taking into account the scales given in the presented results of microscopic analysis, the cavities captured on the cross-sections had sizes varying between about 0.10 μm and 0.25 μm (Figure 3). These pores were significantly smaller compared to the holes on the surfaces of films modified only with essential oil, observed in SEM photographs, whose sizes ranged between about 0.7 μm and 1.0 μm (Figure 1). The regularity of the surfaces of films with the addition of TTO slightly deteriorated as a result of the incorporation of the biocidal substance into the polymer matrix, which is indicated by the increase in the R_a_ roughness parameter from 1.16 μm to 1.74 μm for samples L and LT20, respectively (Figure 3).

Nevertheless, the R_q_ values presented in Figure 3 lead to the conclusion that the micro- and nano-holes are evenly spread on the film surface regardless of the amount of incorporated biocide. The values of the R_max_ parameter confirm that the cavities on the surface of the tested materials are of correspondingly larger size with the increase in the TTO content of the samples (Figure 3). This parameter, in the cases of the neat PLA film and the material with 20% TTO, equaled 14.30 nm and 41.10 nm, respectively. Similar observations of the increase in the roughness of films as a result of the addition of essential oil were made in works devoted to the modification of chitosan with clove essential oil [30] and Carum copticum essential oil [31]. Based on the works of Atarés [32], Javidi [24] and Reyes-Chaparro [30], however, it can be stated that the pores in the external layer of films modified with TTO appeared as a result of the occurrence of the phenomena of the coalescence and creaming of droplets of essential oil inside the polylactide matrix. PLA-based films with the addition of PEG and with or without TTO were also subjected to the AFM analysis.

The fact that the roughness of the film surface consisting of polylactide and poly(ethylene glycol) was greater than the irregularity of the neat polymer sample due to the presence of plasticizer aggregates results from the separation of the PLA and PEG phases [25] (Figure 3). The AFM analysis of the plasticized materials with the addition of tea tree essential oil showed that they had a rougher surface compared to the polylactide and poly(ethylene glycol) film. In addition, the films containing PEG and TTO had pores on the surface similar in size to the pores on the surface of PLA samples modified with TTO (Figure 3). The number of cavities observed in these materials, however, was significantly lower compared to the PLA-TTO systems (Figure 3). Based on the obtained results, it can be concluded that the decrease in the strength of interactions between polylactide chains, caused by the plasticizer applied in the process, impedes the flocculation and coalescence of TTO droplets in the polylactide solution.

### 3.3. Determination of Thermal Properties of PLA-Based Films

The incorporation of both a biocidal substance as well as plasticizer can undoubtedly affect the thermal stability of polymer materials. For this reason, in order to establish the influence of introduced additives on the thermal stability of obtained films, all samples were subjected to thermogravimetric analysis. The temperatures at 5%, 10% and 50% mass loss of all investigated materials (signified as T5%, T10% and T50%, respectively) were listed in Table 1.

**Figure 4:**
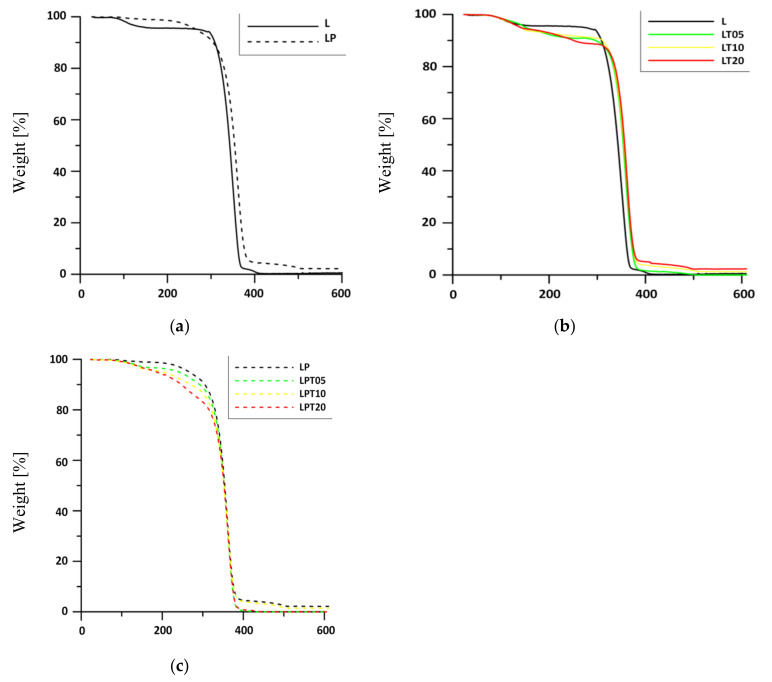
TG curves of (**a**) unplasticized and plasticized PLA film, (**b**) PLA films modified only with TTO and (**c**) PLA based materials with PEG and TTO addition.

The comparison of the thermograms of materials consisting of PLA and PLA with an addition of PEG is shown in Figure 4a.

The thermal degradation of these films took place in two stages [33]. In the range of the lower temperatures (100 °C–250 °C) for L and LP films, there was a comparable mass loss of the sample of about 4.5% of the initial mass. According to the work of Rhim et al. [34], this can be attributed to the evaporation of chloroform residue from the polymer matrix. Nevertheless, the mass of the L sample decreased more significantly in the mentioned temperature range compared to the PLA sample with the addition of the plasticizer. According to the work of Byun [35], the results could suggest a stronger interaction of polylactide chains with volatile substances (including chloroform) after the plasticization of PLA The second stage of the thermal degradation of the PLA film (L) involved the decomposition of polylactide chains. The incorporation of the plasticizer into PLA contributed to a decrease in thermal stability of this material compared to the sample consisting only of polylactide. This occurred as a result of PEG decomposition, which takes place at a much lower temperature than PLA [36]. A reduction in the value of the thermal degradation temperature and a gradual decrease in mass of the PLA sample with the addition of PEG at a temperature below 250 °C was also observed by Phaechamud [33].

In Figure 4b,c, thermograms for the PLA–TTO and PLA–PEG–TTO systems have been presented, respectively. The decomposition of samples modified only with biocide also occurred in two stages, as in the case of materials without the addition of a biocidal substance (Figure 4b). The loss of mass in films modified with TTO at 250 °C ranged from 8.2% in the LT5 film to 9.9% in the LT20 film. The described values of mass loss are higher in comparison to the control film (L), whose mass loss at the mentioned temperature was 5.2%. The registered percentage difference in the decrease of film mass resulted from the presence of essential oil in the PLA matrix.

The incorporation of the plasticizer into the polylactide matrix containing TTO led to a smaller and less violent decrease in the value of T_5%_ compared to films modified only with essential oil. The addition of PEG into the studied materials promoted the loosening of the PLA chains and resulted in the gradual release of volatile substances from the polymer matrix. A regular increase in mass loss can be observed on the thermograms of these films, depending on the increase in the amount of the biocidal substance in particular samples (Figure 4c).

In order to determine the influence of an individual additive on the temperatures and energy effects of the phase transitions of PLA in the obtained films, analyses were carried out using differential scanning calorimetry. The values of thermal properties obtained during studies are listed in Table 1. In the case of a neat PLA sample, the glass transition ($T_{g}^{\mathrm{PLA}}$), cold crystallization ($T_{c}^{\mathrm{PLA}}$) and melting ($T_{m}^{\mathrm{PLA}}$) temperatures reached values equaling 56.5 °C, 124.4 °C and 154.1 °C, respectively (Table 1).

The introduction of tea tree essential oil into the polylactide matrix leads to a significant shift in $T_{g}^{\mathrm{PLA}}$ to lower values (Figure S1). Moreover, the values of the cold crystallization temperature of polylactide in the PLA–TTO films also decreased. Simultaneously, the crystallinity of materials increased with the rise in the content of biocidal substance (Table 1). The above-mentioned phenomena occur due to the presence of the essential oil within the polylactide matrix, as well as the interactions of the polymer with chloroform residues [35]. It should be noted that Qin [14] and Javidi [24] stated that the addition of an essential oil increases the mobility of polylactide chains and leads to their reorganization. On the other hand, according to Byun [35], the presence of chloroform molecules between PLA chains provides conditions preferential for the crystallization of the polymer to take place. Moreover, it should be stressed that the addition of only TTO improved the polymer chain’s movements, facilitating the formation of the crystal structure. The addition of PEG with TTO in all likelihood influenced (increased) the distance between the macromolecules of PLA; for this reason, further increases in *X_c_* were not observed.

Taking into account the samples consisting of PLA, TTO and PEG, it was observed that the incorporation of 5 wt. % PEG into the PLA film reduced the temperatures of all phase transitions (Table 1). It should be stressed that for all the samples containing a plasticizer in the form of PEG, a bimodal peak of melting was observed. This result can be associated with the formation of two crystal forms, as indicated in Tábi’s work [37]. The phenomenon occurred most likely as a result of a localized effect of the plasticizer. In the case of the three-component materials, a significant decrease in the temperatures of phase transition ($T_{c}^{\mathrm{PLA}}$ and $T_{m}^{\mathrm{PLA}}$) was observed compared to the LP results obtained in relation to the samples. Similar observations of a reduction in $T_{c}^{\mathrm{PLA}}$ and $T_{m}^{\mathrm{PLA}}$ were made by Arfat et al. [19] during studies on PLA/PEG films with an addition of clove essential oil. Moreover, the interactions between the chains of PLA, PEG and TTO ingredients allowed us to obtain materials characterized by a low degree of crystallinity compared to samples modified only by means of tea tree essential oil (Table 1).

### 3.4. Evaluation of Mechanical Properties

**Figure 5:**
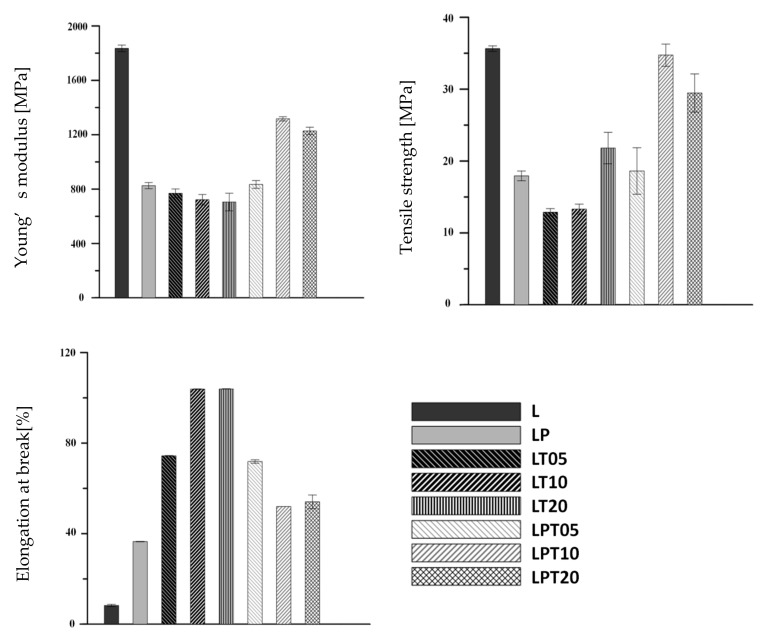
Comparison of mechanical properties of PLA based films.

Due to the potential application of PLA-based materials in packaging, the analysis of the mechanical properties of films is extremely important [14]. The performed studies of the obtained materials resulted in determining such parameters as the Young’s modulus (E), the tensile strength (σ_m_), and the percentage relative elongation at break (ε_b_). Values obtained in relation to the above-mentioned parameters have been presented in Figure 5.

The PLA sample was characterized by high rigidity and resistance to deformation. The value of the Young’s modulus for this film was about 1835.3 MPa, while the maximum stress resistance reached 35.6 ± 0.4 MPa (Figure 5). Due to the brittleness of this material during static stretching, the sample broke at 8.3 ± 0.5% relative elongation. As indicated in other works [25,38], the incorporation of a plasticizer into PLA is intended to increase the flexibility of the obtained materials. According to this statement, an increase in the elasticity of the LP sample, and a simultaneous decrease in the E and σ_m_ compared to the pure PLA film, were observed.

With regard to the results described by Qin [14] and Javidi [24], the susceptibility to deformation increased with the incorporation of essential oil into the polylactide matrix. The film with an addition of 20 wt. % tea tree essential oil, however, had a higher tensile strength compared to other materials modified only with the biocide without the plasticizer. In addition, the samples containing a biocidal substance showed the highest ε_b_ out of all of the obtained films based on polylactide. The plasticizing effect of essential oil is responsible for the increase in the flexibility of the PLA-based materials, which was described in the literature [14,24].The addition of PEG to a PLA solution containing TTO allowed for the obtaining of materials with higher values of Young’s modulus and tensile strength compared to the samples modified with one of the additives (PEG or TTO) (Figure 5).

### 3.5. Thickness and Transparency of Studied PLA-Based Materials

Table 2 depicts the values of thickness as well as transparency, calculated based on the transmittance measurements. In relation to the obtained results, it was established that an addition of essential oil increases the thickness of PLA-based films with respect to the pure PLA film. The same tendency was observed after introducing PEG. The molecules of essential oil and PEG can diffuse between polymer chains and increase the free volume between them, as indicated in the work of Anuar et al. [39]. For this reason, the thickness of the obtained materials was higher compared to a neat polylactide-based film.

The average thickness of the films was used in order to calculate the transparency values according to Equation (2). Observations indicated that an addition of 5% of PEG does not significantly influence the transparency of the LP sample. In the case of samples containing 20% poly(ethylene glycol), described in the work of Arfat [19], the transparency value is higher (*TV* = 2.63) in comparison to the obtained LP film (*TV* = 0.81). This indicates that the transparency of the PLA–PEG materials depends on the composition of the mentioned blends. The other fact which must be taken into account is that the incorporation of tea tree oil into the polylactide, as well as the PLA–PEG system, slightly influences the transparency of the obtained materials. The obtained results suggest that droplets of tea tree oil were evenly distributed in the PLA matrix. For this reason, the extent of the light scattering effect of essential oil droplets in the polymer matrix was not significant [14,19]. Moreover, in the publications of Qin et al. [14], it was established that transparency depends primarily on the types of essential oils.

### 3.6. Examination of Antibacterial Properties

The capacity to combat microorganisms possessed by the obtained PLA films was assessed based on tests carried out by means of the disk-diffusion method. Table 3 summarizes the size of the bacteria-free zones around samples, and the intensity of bacteria proliferation on the surfaces of the samples. According to guidelines included in the ISO 20645:2006 standard, the antibacterial effects of individual materials have been defined as the potential to inhibit the growth of bacteria under conditions favorable for their proliferation (Figures S2 and S3).

Similarly to the method applied in Ahmed’s [11] and Arfat’s [19] research, in relation to a film consisting of neat PLA and PLA with an addition of PEG, used as control samples, the inhibition of the microorganisms’ growth in contact with these materials was not observed. With the increase in the content of tea tree essential oil in PLA films, the antibacterial capacity of samples increased. Nevertheless, in the case of these materials, only a partial inhibition of Gram-positive bacteria was observed. Moreover, the incorporation of 20% TTO into the polymer matrix led to obtaining films with limited antibacterial effects against *S. aureus* and *E. coli*. The reduced capability to combat bacteria displayed by the PLA-based film with an addition of 20% tea tree essential oil, in comparison to other films based on polylactide modified with TTO, results from the premature release of a part of the biocide from the polylactide matrix while the film was being dried. This is due to the coalescence and creaming which occur in the mixture of the PLA solution and TTO [8,18]. The above-mentioned phenomena were observed in SEM photographs (Figure 2) and AFM images (Figure 3).

The incorporation of 5% of the plasticizer into the PLA films with an addition of TTO contributed to the rise in antibacterial potential of the obtained materials. This beneficial change resulted from the effect PEG on PLA, which depends on the reduction of the strength of interactions between polylactide molecules and the increase in PLA-biocide interactions [40]. These materials had enough of the essential oil to inhibit the *S. aureus* and *E. coli* proliferation on their surface. In the case of the film with an addition of PEG and 20% TTO, however, compared to all other materials containing a plasticizer and tea tree essential oil, the area of inhibition of *S. aureus* growth was observed (Table 2). According to the ISO 20645:2006 standard, the incorporation of 20% tea tree essential oil into the PLA/PEG film led to an excessive release of biocidal substances from the polylactide matrix. This also confirms previous conclusions indicating that the processes characteristic of two-phase dispersion systems occurred. In addition, it should be emphasized that regardless of the amount of added tea tree essential oil, all the developed materials containing TTO and the plasticizer were characterized by a positive antibacterial effect against both Gram-positive and Gram-negative bacteria. Based on the obtained results, it was determined that the incorporation of 5% tea tree essential oil and 5% PEG into the PLA matrix allows for obtaining of a film with satisfactory antibacterial properties. The performed analyses also indicate that the addition of PEG into the PLA solution contributed to the increase in the mobility of the polymer chains, which had a direct effect on the release of essential oil from the studied materials [40].

## 4. Conclusions

The studies of films modified only with tea tree essential oil as well as TTO and a plasticizer have shown the beneficial effect of poly(ethylene glycol) on the properties of the obtained materials.

The incorporation of PEG into the PLA matrix, with an addition of tea tree oil, limited the extent of the flocculation and coalescence of the biocidal substance during the drying of the polylactide-based films. In addition, the presence of PEG in PLA samples, modified by means of TTO, led to the increase in the space between the polylactide chains, which contributed to a gradual release of volatile substances. Moreover, it should be stressed that the incorporation of TTO and PEG into PLA contributed to an increase in its resistance to deformation and a rise in its tensile strength values, as well as a significant improvement in the flexibility of the obtained materials, compared to the neat polylactide sample. Furthermore, it can be clearly noted that the incorporation of a plasticizer in the form of poly(ethylene glycol) into PLA, with the addition of TTO, allows one to obtain a material with satisfactory antibacterial properties. In summarizing, this method of modification leads to the formation of bactericidal films based on polylactide with better functional properties compared to those displayed by unmodified PLA samples.

## Figures and Tables

**Table 1:** Thermal properties of PLA-based materials.

Sample	T_5%_ (°C)	T_10%_ (°C)	T_50%_ (°C)	$T_{g}^{PLA}\;({}^{\circ}C)$	$T_{c}^{PLA}\;({}^{\circ}C)$	$∆H_{c}^{PLA}\;(J/g)$	$T_{m}^{PLA}\;({}^{\circ}C)$	$∆H_{m}^{PLA}\;(J/g)$	$X_{c}^{PLA}\;(\%)$
L	273.2	308.7	342.9	56.5	124.4	−12.9	154.1	16.8	3.5
LT05	151.5	296.3	353.9	-	84.0	−11.6	153.3	23.7	11.6
LT10	140.9	307.3	355.2	-	75.8	−10.2	150.9	21.3	11.2
LT20	140.9	251.3	356.7	-	73.6	−9.3	151.3	18.8	10.5
LP	257.2	288.2	338.8	54.9	110.6	−23.0	149.0/154.8	23.6	0.6
LPT05	240.9	292.4	352.8	-	100.6	−23.2	145.0/153.1	24.9	1.6
LPT10	199.5	270.1	352.3	-	98.8	−22.3	144.3/152.1	23.7	1.4
LPT20	185.7	247.3	352.3	-	97.9	−21.9	140.5/150.4	23.0	1.2

T_5%_, T_10%_, T_50%_—temperatures at 5%, 10%, and 50% mass loss. $T_{g}^{\mathrm{PLA}}$, $T_{c}^{\mathrm{PLA}}$, $T_{m}^{\mathrm{PLA}}$—glass transition, cold crystallization and melting temperatures. $∆H_{c}^{PLA}$, $∆H_{m}^{PLA}$—enthalpy of cold crystallization and melting processes. $X_{c}^{PLA}$—degree of crystallinity.

**Table 2:** Thickness and transparency values of PLA-based materials.

Sample	Thickness (mm)	$TV\;({\mathrm{mm}}^{-1})$
L	0.084 ± 0.002	0.75 ± 0.07
LT05	0.087 ± 0.002	0.96 ± 0.02
LT10	0.096 ± 0.004	1.12 ± 0.15
LT20	0.112 ± 0.007	1.33 ± 0.21
LP	0.094 ± 0.003	0.81 ± 0.06
LPT05	0.099 ± 0.004	1.22 ± 0.12
LPT10	0.108 ± 0.006	1.49 ± 0.06
LPT20	0.118 ± 0.005	1.66 ± 0.05

**Table 3:** Results of antibacterial activity of the examined PLA-based films.

Sample	Diameter of Inhibition Zones of Bacteria Growth (mm)		Bacteria Growth in Direct Contact with Sample		Evaluation of Antibacterial Effect ^1^	
	*S. aureus*	*E. coli*	*S. aureus*	*E. coli*	*S. aureus*	*E. coli*
L	0	0	medium	medium	insufficient	insufficient
LP	0	0	medium	medium	insufficient	insufficient
LT05	0	0	weak	lack	limited	good
LT10	0	0	weak	lack	limited	good
LT20	0	0	weak	weak	limited	limited
LPT05	0	0	lack	lack	good	good
LPT10	0	0	lack	lack	good	good
LPT20	1	0	lack	lack	good	good

^1^ in accordance with ISO 20645:2006 standard.

## Supplementary Materials

The following are available online at https://www.mdpi.com/1996-1944/13/21/4953/s1. Figure S1: DSC thermograms of studied PLA-based materials. Figure S2: Photos of Bacteria (*E. coli* and *S. aureus*) growth in direct contact with samples consisted of PLA and TTO. Figure S3: Photos of Bacteria (*E. coli* and *S. aureus*) growth in direct contact with samples consisted of PLA, PEG and TTO.
