# Supplementary material for: Influence of Tea Tree Essential Oil and Poly(ethylene glycol) on Antibacterial and Physicochemical Properties of Polylactide-Based Films
Source: Materials (Basel). 2020 Nov 4;13(21):4953. doi: 10.3390/ma13214953 (PMC7663608; doi:10.3390/ma13214953)
Supplement: Supplementary file 1 [file materials-13-04953-s001.pdf]

materials

MDPI

Supplementary materials

Influence of Tea Tree Essential Oil and Poly(ethylene glycol) on Antibacterial and Physicochemical Properties of Polylactide-Based Films

Iwona Tarach [1], Ewa Olewnik-Kruszkowska [1,*], Agnieszka Richert [2], Magdalena Gierszewska [1] and Anna Rudawska [3]

[1] Chair of Physical Chemistry and Physicochemistry of Polymers, Faculty of Chemistry, Nicolaus Copernicus University in Toruń, Gagarina 7 Street, 87-100 Toruń, Poland; tarach@doktorant.umk.pl (I.T.); mgd@umk.pl (M.G.)

[2] Chair of Genetics, Faculty of Biological and Veterinary Sciences, Nicolaus Copernicus University in Toruń, Lwowska 1 Street, 87-100 Toruń, Poland; a.richert@umk.pl

[3] Department of Production Engineering, Faculty of Mechanical Engineering, Lublin University of Technology, 20-618 Lublin, Poland; a.rudawska@pollub.pl

* Correspondence: olewnik@umk.pl; Tel.: +48-56-611-2210

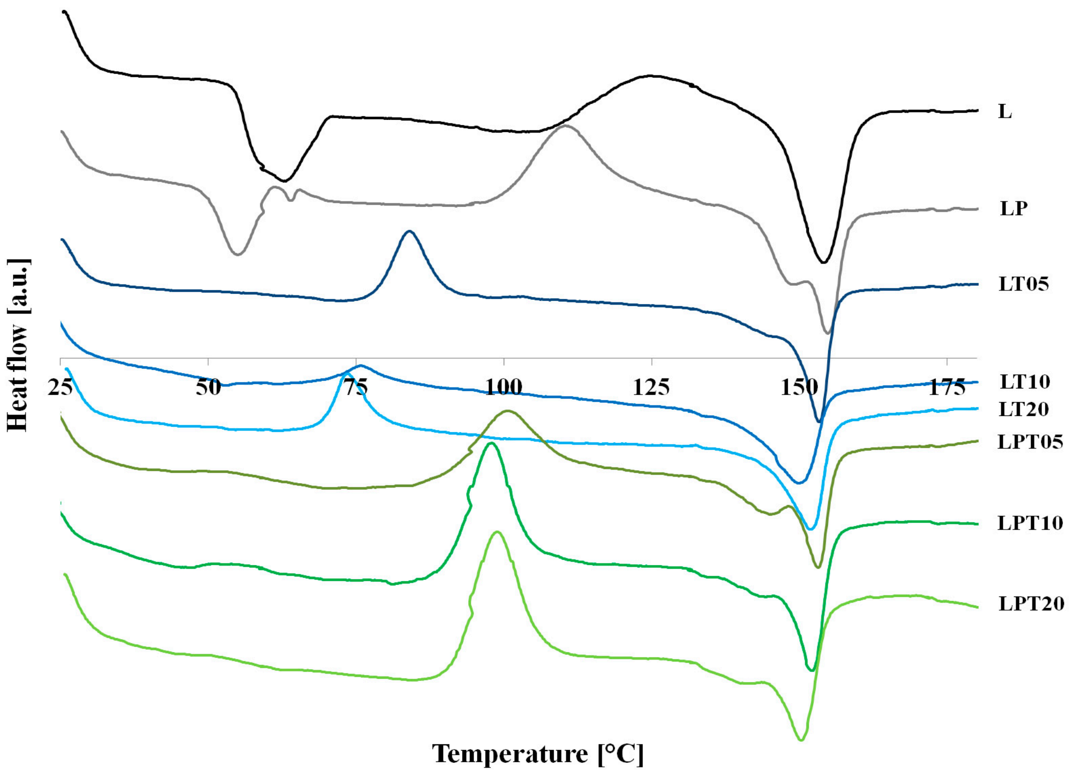

Figure S1. DSC thermograms of studied PLA-based materials

(E. coli)	***(S. aureus)***
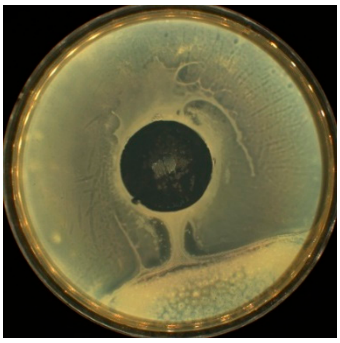	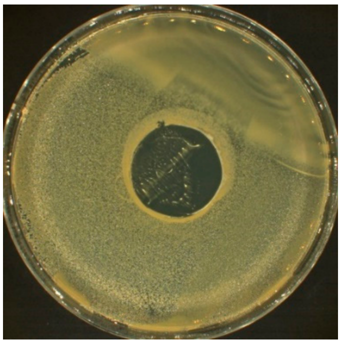
L	L
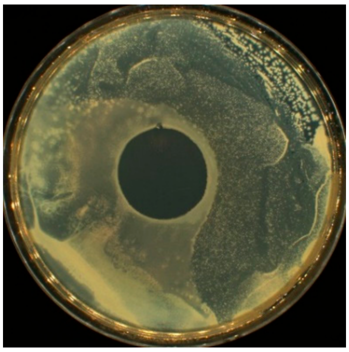	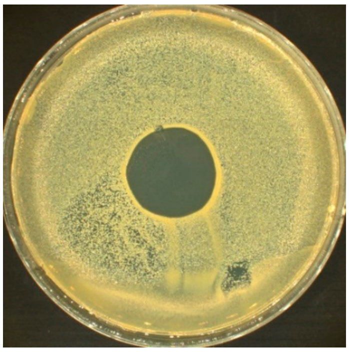
LT05	LT05
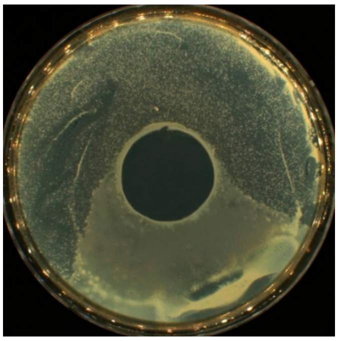	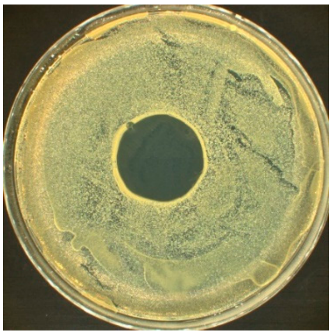
LT10	LT10
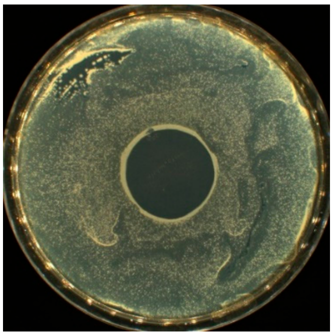	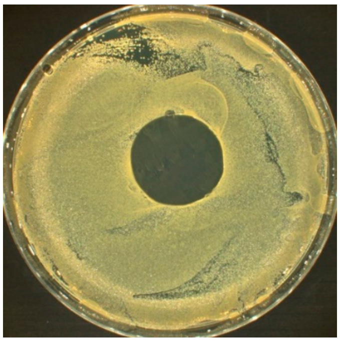
LT20	LT20

Figure S2. Photos of Bacteria (*E. coli* and *S. aureus)* growth in direct contact with samples consisted of PLA and TTO

(E. coli)	***(S. aureus)***

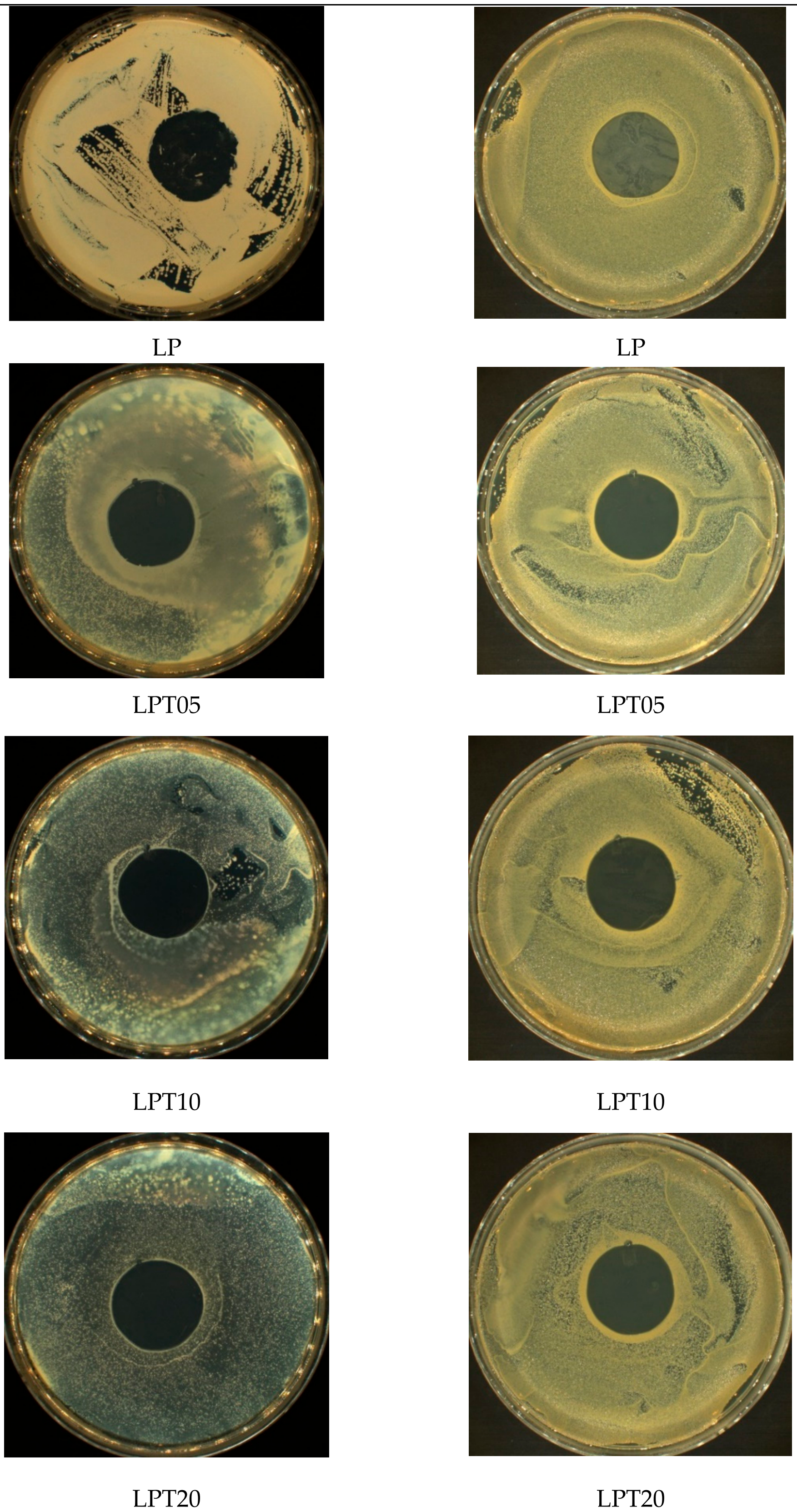

Figure S3. Photos of Bacteria (*E. coli* and *S. aureus*) growth in direct contact with samples consisted of PLA, PEG and TTO.
